# Nac1 interacts with the POZ-domain transcription factor, Miz1

**DOI:** 10.1042/BSR20140049

**Published:** 2014-06-05

**Authors:** Mark A. Stead, Stephanie C. Wright

**Affiliations:** *School of Biology, University of Leeds, Leeds LS2 9JT, U.K.

**Keywords:** BTB domain, Miz1, Nac1, ovarian cancer, POZ domain, transcriptional repressor, BEN, B-cell translocation gene 3 associated nuclear protein, E5R and Nac1, BMB, 1,4-bismaleimidobutane, BTB, bric-à-brac, tramtrack and broad complex, FASN, fatty acid synthase, FLAG-Nac1^2–514^, FLAG-tagged Nac1, *Gadd45GIP1*, growth arrest and DNA-damage-inducible 45-γ interacting protein, GFP-Miz1^2–794^, green fluorescent protein-tagged full-length Miz1, HA, haemagglutinin, HA-Miz1^2–794^, HA-tagged Miz1, mCherry-Nac1^2–794^, mCherry-tagged full-length Nac1, Miz1, Myc-interacting zinc-finger protein 1, Nac1, nucleus accumbens 1, POZ, poxvirus and zinc finger, POZ-TF, POZ-domain transcription factor, siRNA, small interfering RNA

## Abstract

Nac1 (nucleus accumbens 1) is a POZ (poxvirus and zinc finger)-domain transcriptional repressor that is expressed at high levels in ovarian serous carcinoma. Here we identify Nac1 as a novel interacting partner of the POZ-domain transcriptional activator, Miz1 (Myc-interacting zinc-finger protein 1), and using chemical crosslinking we show that this association is mediated by a heterodimeric interaction of the Nac1 and Miz1 POZ domains. Nac1 is found in discrete bodies within the nucleus of mammalian cells, and we demonstrate the relocalization of Miz1 to these structures in transfected HeLa cells. We show that siRNA (small interfering RNA)-mediated knockdown of Nac1 in ovarian cancer cells results in increased levels of the Miz1 target gene product, p21^Cip1^. The interaction of Nac1 with Miz1 may thus be relevant to its mechanism of tumourigenesis in ovarian cancer.

## Abstract 2

Nac1 is a transcriptional repressor that has been implicated in ovarian serous carcinoma. Here we show that Nac1 interacts with the transcription factor Miz1, and suggest that this interaction may contribute to tumourigenesis.

## INTRODUCTION

Nac1 (nucleus accumbens 1) was originally identified as the protein product of a cocaine-inducible transcript in the nucleus accumbens of the rat brain [1], and more recently it has emerged as a transcriptional repressor that functions as part of the network involved in embryonic stem cell self-renewal [2]. Amplification of the gene encoding Nac1, *NACC1*, has been implicated as one of the top potential ‘drivers’ in human ovarian serous carcinoma [3], and high levels of Nac1 may also be relevant in ovarian clear cell carcinoma [4] and in some cervical [5] and uterine [6] cancers. Elevated Nac1 levels in ovarian serous carcinoma are particularly common in drug-resistant disease that is associated with relapse following initial treatment [7,8]. The artificial knockdown or inactivation of Nac1 leads to the apoptosis of Nac1-overexpressing ovarian cancer cells [7] and restores their sensitivity to chemotherapeutics [9], thereby validating this protein as a potential therapeutic target.

A variety of mechanisms are involved in Nac1-mediated tumourigenesis and drug-resistance. Nac1 induces cell proliferation in part by repressing transcription of the *Gadd45GIP1* (growth arrest and DNA-damage-inducible 45-γ interacting protein) gene [10] and negative regulation of the Gadd45 pathway also contrib-utes to paclitaxel-resistance [11]. Elevated Nac1 also contributes to tumour aggressiveness and drug-resistance by increasing the levels of FASN (fatty acid synthase) and thereby modulating fatty acid metabolism. Treatment failure in ovarian cancer is associated with drug-induced activation of HMGB1-mediated autophagy and with the inhibition of senescence, and Nac1 plays an essential role in both of these processes [12,13]. It has recently been shown that Nac1 also has non-nuclear functions, and interacts with monomeric actin to promote cytokinesis in highly proliferating Nac1-overexpressing cancer cells [14].

Nac1 is a POZ-TF [POZ (poxvirus and zinc finger); also known as BTB (bric-à-brac, tramtrack and broad complex)-domain transcription factor] that functions as a repressor in both neuronal and non-neuronal cells [15,16]. Transcription factor POZ domains serve to recruit co-repressors and also mediate dimerization and heteromeric interactions between different POZ-TFs (reviewed in [17]); the Nac1 POZ domain is a classic POZ-domain dimer [18], and interacts with the corepressor COREST [19] and with the histone deacetylases HDAC3 and HDAC4 [16]. POZ domains are also found in adaptor proteins that recruit substrates for ubiquitination by the cullin-type E3 ligase complexes; these adaptors interact with cullin3 via their POZ-domain, and they recruit substrates via additional domains such as kelch, MATH or zinc fingers. It has recently been recognized that some transcription factor POZ domains can also interact with cullin3 [20,21] and thereby play a role in regulating the ubiquitination of their corepressors and other interacting partners [22,23]. Nac1 interacts with cullin3 in neuronal cells [24], although its role in ubiquitination has not been characterized. Target genes of Nac1 have been identified in embryonic stem cells [25], but the mechanism whereby Nac1 interacts with DNA is not known. Nac1 does not contain a zinc-finger DNA-binding domain as is found in most POZ-TFs; however, it is assumed that its C-terminal BEN (B-cell translocation gene 3 associated nuclear protein, E5R and Nac1) domain interacts with the promoters of target genes as has been recently reported for other BEN-domain transcriptional repressors [26]. Many POZ-domain transcription factors and adaptors are localized within discrete structures in the nucleus [27], and Nac1 is found in nuclear bodies, termed Nac1 bodies, at specific stages of the cell cycle [28].

Miz1 (Myc-interacting zinc-finger protein 1) is a POZ-TF that was originally identified as a c-Myc interacting protein [29] in yeast two-hybrid protein interaction screens. Miz1 regulates genes involved in growth arrest [30,31], differentiation [32–34], apoptosis [35] cell adhesion [36] and autophagy [37], and plays a central role in DNA-damage responses [38–41], lymphoid development [42–45] and inflammation [46]. Miz1 acts as a transcriptional activator via the recruitment of the cofactors p300 [31] and nucleophosmin [47], and it binds to the initiator DNA element of target gene promoters via its central zinc-finger DNA-binding domain. The transcriptional properties of Miz1 are altered by its interaction with other transcription factors, and it acts as a repressor when in complex with c-Myc [29], BCL6 [48] or Zbtb4 [49]. Miz1 target genes include the cell cycle inhibitors *CDKN1A* [39] and *CDKN2B* [30,31], the differentiation-associated *Mad4* gene [33], the anti-apoptotic *Bcl2* gene [50], the autophagy gene *Ambra1* [37], and the *alpha6* and *beta1 integrin* genes involved in cell adhesion [36]. The repression of Miz1 target genes by Miz1/c-Myc and by Miz1/BCL6 complexes is important in physiological apoptotic responses [35] and in B-cell development [48] respectively; however, the inappropriate repression of cell-cycle inhibitors contributes to deregulated proliferation in tumours associated with the overexpression of c-Myc [51,52] or BCL6 [48]. Miz1 interacts with c-Myc via residues adjacent to its DNA-binding domain [29], and with Zbtb4 and BCL6 via its N-terminal POZ domain [48,49]. Although most transcription factor POZ domains are dimeric [17], the Miz1 POZ domain forms both dimers [53] and tetramers *in vitro* [54]; importantly, the stoichiometry of heteromeric Miz1 POZ domain interactions has not been elucidated.

In this report, we identify Nac1 as a novel interacting partner of Miz1, and show that this interaction is mediated by a heterodimeric association between the POZ domains of these proteins. The interaction of Nac1 with Miz1 leads to the recruitment of Miz1 into Nac1 nuclear bodies, and the artificial knock-down of Nac1 in an ovarian cancer cell line results in increased levels of the Miz-1 target gene product, p21^Cip1^. The interaction between Nac1 and Miz1 may thus contribute to tumourigenesis in Nac1-overexpressing ovarian cancer cells, analogous to the role of the BCL6/Miz1 interaction in diffuse large cell B-cell lymphoma.

## EXPERIMENTAL

### Antibodies

Rabbit anti-FLAG polyclonal (Sigma F7425), rabbit anti-HA (haemagglutinin) polyclonal (Santa Cruz Y-11 [sc-805]), rabbit anti-p21 polyclonal (Abcam ab7960), mouse anti-Nac1 monoclonal (Abcam ab81987) and mouse anti-GAPDH monoclonal (Calbiochem CB1001) antibodies were used in western blots at 0.5, 2, 5, 0.82 and 1 μg/ml, respectively. Horseradish peroxidase-conjugated goat anti-rabbit IgG and goat anti-mouse IgG secondary antibodies (Pierce 31460 and 31430, respectively) were used at 40 ng/ml.

### Cloning

Mouse Miz1 and Nac1 cDNAs were amplified from an embryonic stem cell cDNA library using Phusion high-fidelity DNA polymerase (Thermo Fisher) and inserted into a vector that expresses fusion proteins containing an N-terminal 3× FLAG or 3× HA tag under control of the CMV (cytomegalovirus) promoter. cDNAs encoding POZ domains were also expressed as fusion proteins that additionally contain a C-terminal nuclear localization signal (mouse c-Myc residues 353–361). Miz1 and Nac1 cDNAs were cloned into pEGFP-C1 or pmCherry-C1 (Clontech Laboratories Inc.) for the expression of fluorescently tagged proteins. Site-directed mutagenesis was carried out by PCR using Phusion high-fidelity DNA polymerase.

### Yeast two-hybrid assays

Yeast two-hybrid assays were performed using *Saccharomyces cerevisiae* AH109 transformed with 200 ng of each plasmid DNA as described in [55]. The mouse Miz1 POZ domain (residues 1–115) was expressed as a GAL4 activation domain fusion using the vector pGADT7 (Clontech Laboratories Inc.), and the POZ domains of 32 mouse transcription factors were expressed as GAL4 DNA-binding domain fusions using pGBKT7 (Clontech Laboratories Inc.).

### Transfection of mammalian cell lines

HeLa and COS-7 cells were cultured in Eagle's minimal essential medium (alpha modification) supplemented with 10% (v/v) FBS, 50 units/ml penicillin and 50 μg/ml streptomycin at 37°C and 5% (v/v) CO_2_. COS-7 cells for co-immunoprecipitation experiments were seeded into 90-mm-diameter dishes and transfected with a total of 40 μg DNA using the calcium phosphate method [56]; cells were harvested 36 h post-transfection. HeLa cells for fluorescence microscopy experiments were seeded onto glass slides in 35-mm-diameter dishes and transfected with a total of 1 μg DNA using GeneJuice® Transfection Reagent (Novagen).

### Co-immunoprecipitation using anti-FLAG resin

Approximately 10^7^ cells were rinsed with PBS, pelleted at 1500 ***g***, and resuspended in 200 μl lysis buffer [50 mM Tris–HCl (pH 7.4), 150 mM NaCl, 1 mM EDTA, 1% (v/v) Triton X-100]. Samples were incubated on ice for 30 min and the lysate clarified by centrifugation at 12000 ***g*** for 10 min at 4°C. Clarified lysate was added to 20 μl TBS-equilibrated [50 mM Tris–HCl (pH 7.4), 150 mM NaCl] anti-FLAG M2 affinity gel (Sigma) and incubated for 5 h at 4°C. The resin was pelleted by centrifugation at 7000 ***g*** for 1 min at 4°C, and washed three times with 1 ml TBS. The samples were boiled with SDS-sample buffer [125 mM Tris–HCl (pH 6.8), 4% (w/v) SDS, 20% (v/v) glycerol, 0.1% (w/v) bromophenol blue] and analysed by western blotting.

### Thiol crosslinking *in vivo*

48 h post-transfection, HeLa cells were rinsed twice in PBS and incubated for 5 min at 37°C in Kreb’s-Ringer phosphate buffer [118 mM NaCl, 4.7 mM KCl, 2.5 mM CaCl_2_, 1.2 mM KH_2_PO_4_, 1.2 mM MgSO_4_, 16.2 mM Na_2_HPO_4_ (pH 7.4)]. Cells were incubated at 4°C for 5 min and BMB (1,4-bismaleimidobutane) was added to a final concentration of 100 μM. Crosslinking was performed at 4°C for 10 min and the reaction was quenched by the addition of L-cysteine to 4 mM [57]. Cells were rinsed with PBS, pelleted at 1500 ***g***, resuspended in SDS-sample buffer containing 10% (v/v) 2-mercaptoethanol, and analysed by western blotting.

### Transfection of A2780 cells with siRNA (small interfering RNA)

A2780 ovarian cancer cells were cultured in RPMI 1640 medium supplemented with 10% FBS at 37°C and 5% (v/v) CO_2_ and seeded into 6-well plates at a density of 1 × 10^5^ cells per well. Cells were incubated for 24 h and the medium replaced with OPTI-MEM-reduced serum medium (Invitrogen). 175 pmol ON-TARGETplus SMARTpool siRNA (Dharmacon; target sequences: CGGCUGAACUUAUCAACCA, GGGCGCAGCUGAUGAACUG, GGGCAUGGAUGAGCAGUAC, CGAAAUCGCAUCCGGGUUC) was delivered into the cells using 35 μl Oligofectamine (Invitrogen) as described in the manufacturers protocol. Control cells were treated with 175 pmol non-targeting ON-TARGETplus siRNA (Dharmacon). Cells were incubated for 4 h at 37°C/5% CO_2_ before being supplemented with FBS to a final concentration of 10%. Cells were harvested 48 h post-transfection, boiled in SDS-sample buffer containing 10% (v/v) 2-mercaptoethanol and analysed by western blotting.

### Fluorescence microscopy

Fluorescence microscopy was carried out using the DeltaVision Optical Restoration Microscopy System (Applied Precision Inc.). Images were collected from 50 × 0.2 μm thick optical sections, and 3D datasets were deconvoluted using the default settings on the softWoRx deconvolution algorithm (Applied Precision Inc.).

## RESULTS AND DISCUSSION

### Miz1 interacts with Nac1 in yeast two-hybrid assays and in mammalian cells

**Figure 1:**
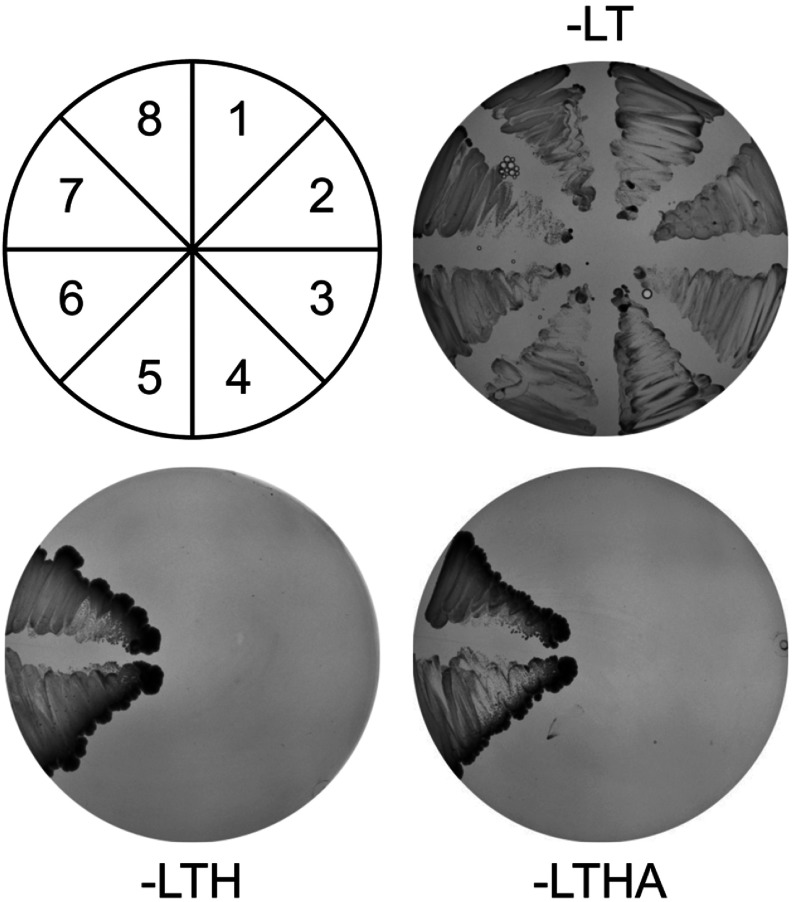
Interaction of the Nac1 and Miz1 POZ domains in yeast two-hybrid assays AH109 yeast cells were transformed with constructs expressing GAL4 activation domain fusion proteins (pGADT7) together with GAL4 DNA-binding domain fusion proteins (pGBKT7) and plated onto media lacking leucine and tryptophan [-LT], lacking leucine, tryptophan and histidine [-HLT], and lacking leucine, tryptophan, histidine and adenine [-LTHA]. Transformation with Miz1POZ-pGADT7 together with: (1) pGBKT7, (2) Zbtb8 POZ-pGBKT7, (3) Zbtb6 POZ-pGBKT7, (6) Nac1 POZ-pGBKT7, and (7) BCL6 POZ-pGBKT7 (7). Transformation with Nac1 POZ-pGBKT7 together with: (4) Zbtb8 POZ-pGADT7, (5) Zbtb6-pGADT7 and (8) pGADT7.

The transcriptional properties of Miz1 may be modulated by the interaction of its N-terminal POZ domain with other POZ domain transcription factors [48]. To identify novel interacting partners of Miz1, we used yeast two-hybrid assays to analyse the interaction of its POZ domain (Miz1 residues 1–115) with the POZ domains isolated from 32 POZ-TFs; the well-characterized interaction between the Miz1- and BCL6 POZ domains served as a positive control. The Miz1 POZ domain interacted strongly with the POZ domain of the transcriptional repressor, Nac1, in these assays (Figure 1, panel 6).

**Figure 2:**
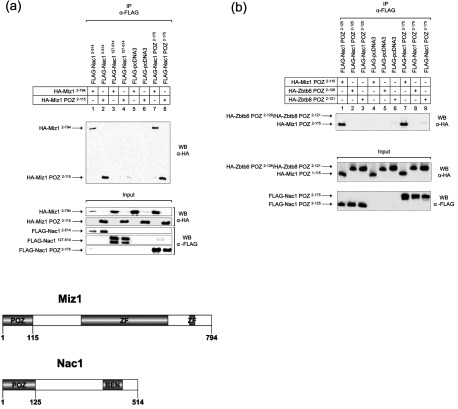
Co-immunoprecipitation of Nac1 and Miz1 in transfected HeLa cells (a) HeLa cells were transfected with FLAG-tagged Nac1 and HA-tagged Miz1 constructs. Lysates were immunoprecipitated using anti-FLAG resin, and samples analysed by western blotting using anti-HA antibody. (b) HeLa cells were transfected with FLAG-tagged Nac1 POZ domain constructs together with constructs that expressed the Miz1, Zbtb8 and Zbtb6 POZ domains.

To determine whether Nac1 interacts with Miz1 in mammalian cells, we expressed the full-length proteins, FLAG-Nac1^2–514^ (FLAG-tagged Nac1) and HA-Miz1^2–794^ (HA-tagged Miz1), in COS-7 cells and analysed their interaction by co-immunoprecipitation using anti-FLAG resin; we also analysed the interaction between FLAG-Nac1^2–514^ and a truncated protein comprising the Miz1 POZ domain only (HA-Miz1 POZ^2–115^). FLAG-Nac1^2–514^ interacted both with HA-Miz1^2–794^ and with HA-Miz1 POZ^2–115^ in these assays (Figure 2a, lanes 1 and 2), and deletion of the Nac1 POZ domain (FLAG-Nac1^127–514^) abolished these associations (Figure 2a, lanes 3 and 4). We also expressed the N-terminal POZ domain of Nac1 as a FLAG-tagged protein (FLAG-Nac1^2–175^), and demonstrated its interaction both with HA-Miz1^2–794^ and with HA-Miz1 POZ^2–115^ (Figure 2a, lanes 7 and 8).

To confirm the specificity of the interaction between the Nac1 and Miz1 POZ domains in mammalian cells, we analysed the interaction of the Nac1 POZ domain with the POZ domains of the zinc-finger proteins Zbtb6 and Zbtb8; these POZ domains did not interact with Nac1 in yeast two-hybrid assays (Figure 1). The Nac1 POZ domain was expressed as two FLAG-tagged proteins, FLAG-Nac1^2–125^ and FLAG-Nac1^2–175^, both of which interacted with the Miz1 POZ domain, but not with Zbtb6 or Zbtb8 (Figure 2b).

### The interaction between Nac1 and Miz1 leads to the relocalization of Miz1 into Nac1 nuclear bodies

**Figure 3:**
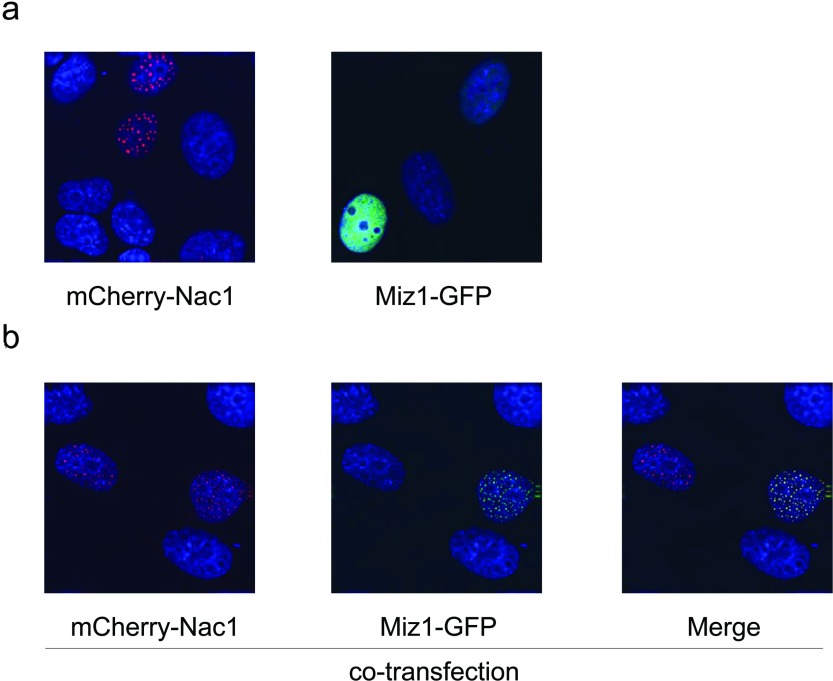
The interaction between Nac1 and Miz1 leads to the relocalization of Miz1 into Nac1 nuclear bodies HeLa cells were transfected with constructs that expressed EGFP (enhanced GFP)-tagged and pmCherry-tagged proteins as indicated.

The nucleus contains distinct nuclear bodies that compartmentalize the organelle to facilitate efficient biological processes (reviewed in [58]). Several POZ-domain transcription factors and Cul3 adaptors are found in discrete nuclear structures that have variously been termed bodies or speckles [27], and the localization of these POZ proteins in nuclear bodies directs the co-localization of their interacting partners [23,59]. Nac1 is found in discrete bodies within the nucleus of normal and cancer cells [7]; although it has not been determined whether these structures play a role in transcriptional regulation or protein ubiquitination, it is conceivable that they could represent hubs that recruit and silence multiple specific gene loci in a manner similar to the repressive function of polycomb bodies (reviewed in [58]). In order to determine whether the subcellular localization of Miz1 is modulated by its interaction with Nac1, we expressed fluorescently tagged Miz1 and Nac1 proteins in HeLa cells. When expressed individually, mCherry-Nac1^2–794^ (mCherry-tagged full-length Nac1) was found in nuclear bodies, consistent with previous reports, whereas GFP-Miz1^2–794^ (green fluorescent protein-tagged full-length Miz1) showed diffuse nuclear flourescence (Figure 3a). The co-expression of GFP-Miz1^2–794^ with mCherry-Nac1^2–794^ led to its relocalization into Nac1 nuclear bodies (Figure 3b).

### siRNA-mediated knock-down of Nac1 leads to the induction of the Miz1 target gene product, p21^Cip1^, in ovarian cancer cells

**Figure 4:**
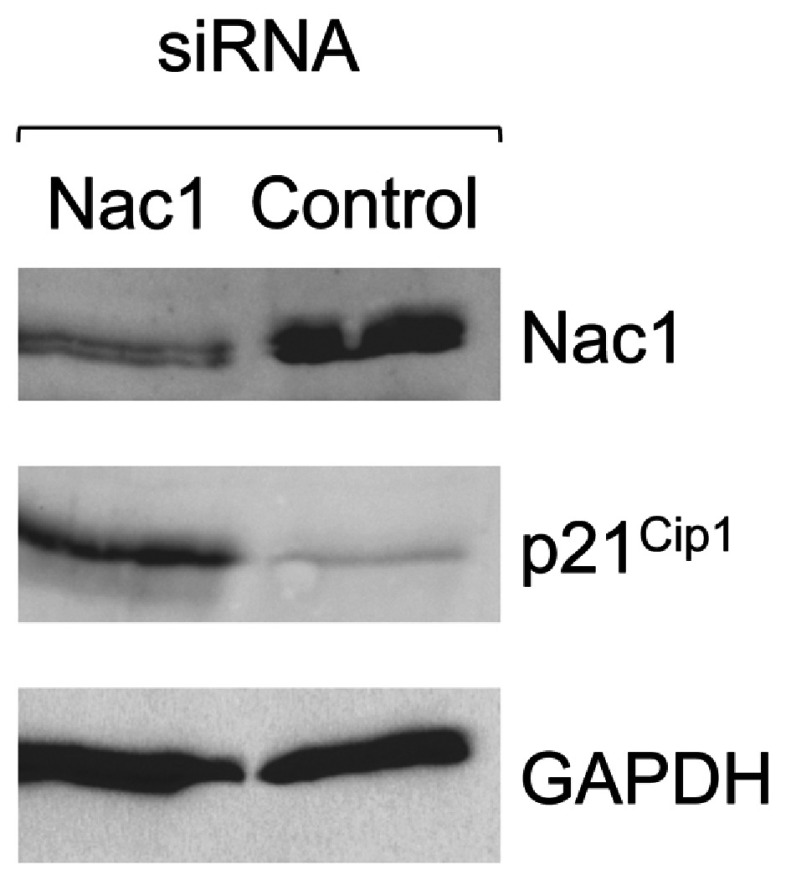
siRNA-mediated knockdown of Nac1 leads to the induction of p21^Cip1^ in A2780 ovarian carcinoma cells A2780 cells were treated with siRNA that targets Nac1 or with control siRNA, and levels of Nac1, p21Cip1 and GAPDH were measured by western blotting.

The interaction of Miz1 with the transcription factors c-Myc or BCL6 leads to the suppression of Miz1 target genes associated with growth arrest and differentiation [30,31,39,48], and this may contribute to aberrant growth control in malignancies associated with the overexpression of these oncoproteins. Nac1is overexpressed in advanced ovarian serous carcinoma and is found at high levels in the human ovarian cancer cell line, A2780. We therefore determined the effect of siRNA-mediated knock-down of Nac1 on levels of the p21^Cip1^ protein product of the Miz1 target gene, *CDKN1A*, in A2780 cells. Treatment of A2780 ovarian cancer cells with an siRNA that targets Nac1 led to a 2.6-fold reduction in Nac1 levels, and a concomitant 7.7-fold increase in levels of p21^Cip1^ (Figure 4), consistent with repression of *CDKN1A* gene by the Nac1/Miz1 complex in these cells.

### The Nac1 and Miz1 POZ domains form a heterodimeric complex

Transcription factor POZ domains are obligate domain-swapped dimers that have an extensive hydrophobic interface that mainly comprises residues from the N-terminal α helices of each chain [60,61] (reviewed in [17]); a tetrameric association of the Miz1 POZ domain has also been observed [54]. Crucially, the stoichiometry of heteromeric POZ domain interactions has not been experimentally determined, and we therefore attempted to determine whether the Miz1- and Nac1 POZ domains are capable of forming a heterodimeric species.

**Figure 5:**
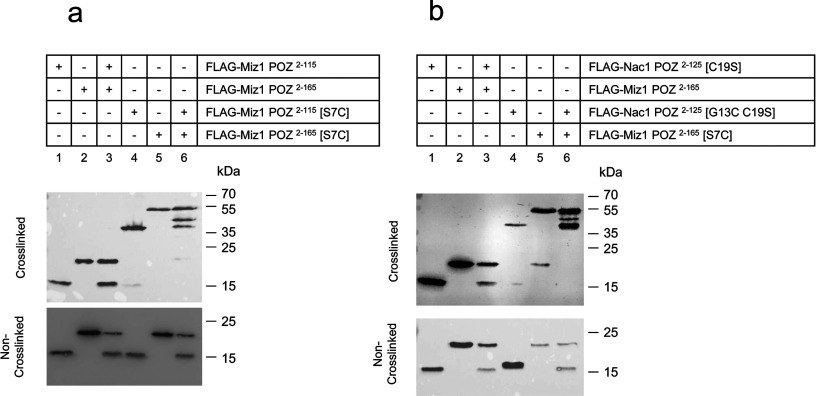
The Nac1 and Miz1 POZ domains form a heterodimeric species when expressed in COS-7 cells (a) COS-7 cells were transfected with FLAG-tagged wild-type and mutant Miz1 POZ domains and treated with BMB. Lysates were analysed by western blotting using anti-FLAG antibody. (b) COS-7 cells were transfected with FLAG-tagged Nac1 and Miz1 POZ domains and treated as in (a).

The POZ domain transcription factor, Bach2, contains a cysteine residue (Cys^20^) located in the N-terminal alpha helix (α1) of the POZ domain, and crystal structures of the Bach2 POZ domain dimer revealed an inter-subunit disulphide bond between the Cys^20^ residues of the two chains (PDB entries 3ohu and 3ohv [62]). We used crystal structures of the Nac1 and Miz1 POZ domains (PDB entries 3ga1 and 2q81) to model a cysteine residue in place of the residue (Nac1 Gly^13^ and Miz1 Ser^7^) corresponding to Bach2 Cys^20^; the proximity of the cysteine residues in the mutant POZ domain models suggested that Nac1^[G13C]^ and Miz1^[S7C]^ would each be capable of forming disulphide-bonded homodimers. We also mutated the Cys^19^ residue of the Nac1 POZ domain α1 helix in order to prevent potential spurious disulphide bond formation between nearby cysteine residues; this cysteine residue is not conserved among the POZ domains of other proteins. Mutant Nac1 and Miz1 POZ domains, Nac1^[G13C C19S]^ and Miz1^[S7C]^, were expressed as FLAG-tagged proteins in COS-7 cells; the Miz1 POZ domain was expressed as two forms of FLAG-tagged protein (FLAG-Miz1 POZ^2–115^ and FLAG-Miz1 POZ^2–165^) that were readily distinguishable in size, and the Nac1 POZ domain was expressed as FLAG-Nac1 POZ^2–125^. As expected, neither of the wild-type Miz1 POZ domains, FLAG-Miz1 POZ^2–115^ or FLAG-Miz1 POZ^2–165^, formed disulphide-bonded species when expressed in COS-7 cells and treated with the thiol crosslinking reagent BMB (Figure 5a, lanes 1–3). In contrast, the BMB-treated mutant Miz1 POZ domains, FLAG-Miz1 POZ^2–115 [S7C]^ and FLAG-Miz1 POZ^2–165 [S7C]^, each formed disulphide-bonded dimers when expressed individually (Figure 5a, lanes 4 and 5), and when expressed together produced an additional species consistent with heterodimerization between the two proteins (Figure 5a, lane 6). Similarly, the wild-type Nac1 POZ domain, FLAG-Nac1 POZ^2–125^, was unable to form a disulphide-bonded dimer when expressed in COS-7 cells either alone or with the wild-type FLAG-Miz1 POZ^2–165^ (Figure 5b, lanes 1 and 3). The mutant FLAG-Nac1 POZ^2–125 [G13C C19S]^ formed a disulphide-bonded dimer (Figure 5b, lane 4) when expressed alone in COS-7 cells, and when FLAG-Nac1 POZ^2–125 [G13C C19S]^ and FLAG-Miz1 POZ^2–165 [S7C]^ were expressed together and treated with BMB, an additional species was produced that was consistent with heterodimerization between the Nac1 and Miz1 POZ domains (Figure 5b, lane 6).

## CONCLUSION

Identification of the Miz1/Nac1 interaction expands the repertoire of interactions involving the Miz1 POZ domain and may have relevance to the mechanism of tumourigenesis by Nac1 in ovarian cancer. The recent identification of Nac1 as a direct target gene that is activated by Miz1 [37] may also suggest that the Miz1/Nac1 interaction could contribute to the autoregulation of Nac1 expression and of autophagy. The interaction between the Nac1 and Miz1 POZ domains represents a heterodimeric association and it will be relevant to determine both the stoichiometry of other heteromeric POZ domain interactions and the features that determine their specificity.
